# An Evaluation Method for the Suppression of Pathogenic *Fusarium oxysporum* by Soil Microorganisms Using the Dilution Plate Technique

**DOI:** 10.1264/jsme2.ME16052

**Published:** 2016-08-24

**Authors:** Masahiro Mitsuboshi, Yuuzou Kioka, Katsunori Noguchi, Susumu Asakawa

**Affiliations:** 1 Tsukuba Research Institute, Katakura & Co-op Agri Corporation5–5511 Namiki, Tsuchiura 300–0061Japan; 2 Katakura & Co-op Agri Corporation1–8–10 Kudankita, Chiyoda, Tokyo 102–0073Japan; 3 Graduate School of Bioagricultural Sciences, Nagoya University1 Furo-cho, Chikusa, Nagoya 464–8601Japan

**Keywords:** Spinach, culture technique, biological diagnosis, *Fusarium oxysporum*, organic fertilizer

## Abstract

Soil-borne diseases caused by pathogenic microorganisms are one of the main factors responsible for the decline in crop yields in farmlands. Pathogenic *Fusarium oxysporum* causes serious damage to various crops, and, thus, a feasible diagnostic method for soil-borne diseases is required. We herein examined a simple method to evaluate the suppressiveness of soil microorganisms against a pathogen by co-cultivating indigenous soil microorganisms and a pathogenic fungus (*F. oxysporum* f. sp. *spinaciae*). We inoculated *F. oxysporum* onto the center of agar medium plates mixed with a dilution series of a suspension of organic fertilizers or soil. After an approximately one-week cultivation, the growth degree of *F. oxysporum* was estimated based on the size of the colonies that formed on the plates. The growth degree of *F. oxysporum* significantly differed among the organic fertilizers tested, indicating the usefulness of the method for evaluating suppressiveness by organic fertilizers. Differences in the growth degrees of *F. oxysporum* were associated with the incidence of disease in spinach on soil treated with organic fertilizers and inoculated with a pathogenic *F. oxysporum* strain. These results suggested that this method provides some useful information on the suppressiveness of organic fertilizers and soil against *Fusarium* wilt.

Soil-borne diseases are one of the main factors responsible for the decline in crop yields in farmlands. Some soils suppress soil-borne diseases, even those with a high population density of pathogens, and are known as “suppressive soil”. Not only the physicochemical, but also the biological characteristics of soil are associated with suppressiveness (11, 16). The quality of soil is measured by a combination of its physical, chemical, and biological characteristics (4). Organic fertilizers induce suppressiveness by influencing the composition and activity of soil microbial communities (5). Liu *et al.* (14) previously suggested that enhancements in soil suppressiveness against bacterial wilt by organic fertilizers are induced by multiple factors, particularly alterations in the physicochemical and biological properties of soil.

The enumeration of microbial populations, assays of microbial activities, and estimation of antagonism toward pathogens represent potential diagnostic measurements for the microbiological characteristics of soil. Viable counts of pathogens and the ratio of viable counts of bacteria to fungi in soil are used to evaluate the incidence of soil-borne diseases (18). Bruggen *et al.* (6) measured fluctuations in the viable number of soil microbes after the application of compost to soil and found that the incidence of *Fusarium* disease was suppressed when the amplitude of the fluctuation was low. However, difficulties are associated with evaluating the functions of the soil microbial community only via the enumeration of the viable number of microbes (22). Besides the viable counts of microbial populations, many analytical methods such as microbial biomass carbon (2), a phospholipid fatty acid (PLFA) analysis, and community level substrate utilization (CLSU) profiling have been used and enabled the grouping of soils, but did not show a clear relationship with the suppressiveness of soil-borne diseases (21).

Techniques based on DNA analyses are used to evaluate the species composition of soil microbial communities (10). A difference in the species compositions of bacteria and archaea was found in the rhizosphere microbiome of sugar beet between suppressive and conducive soils (15). Klein *et al.* (13) reported shifts in the root microbiome in soil suppressive to a *Fusarium* disease. Urashima *et al.* (20) demonstrated using a PCR-DGGE analysis of the *Fusarium* community structure that the proportions of the band related to pathogenic *F. oxysporum* to asparagus were higher in growthinhibited fields than in fields without growth inhibition, and also showed the applicability of this analysis to the biological diagnosis of asparagus fields. In an analysis of the factors associated with natural soil suppressiveness against potato common scab, the copy number of the *txtB* gene in the periderm was significantly lower in suppressive soil than in conducive soil (19). These findings indicate that the composition and abundance of microbes differ between suppressive and conducive soils. Techniques using DNA analyses may easily reveal the community composition and abundance of microbes present in soil. However, the functions of the microbial community related to their composition and abundance cannot be evaluated. For example, soil suppressiveness against a soilborne disease cannot be estimated from the abundance of the respective pathogen only because suppressive activities by other indigenous microbes in soil are not considered. In addition, DNA-based techniques are commonly used in research organizations, but some difficulties are still associated with its applicability to general farmers even via agricultural extension centers.

Cha *et al.* (7) reported the usefulness of a combination of molecular and culture techniques to reveal the microbial and biochemical bases of suppressive effects in protective soil against strawberry *Fusarium* wilt. Regarding antagonism, Alam *et al.* (1) found that a fungal strain exerting a plant growth-promoting effect also exhibited antagonism against *Fusarium* wilt disease and suppressed the incidence of the disease following its inoculation into soil. Hashimoto *et al.* (9) developed a microbial biosensor as a simple and rapid diagnostic tool for soil-borne diseases and applied it to an estimation of the adaptation of antagonists to disease-infested soil. However, a method to directly measure the degree of suppressive effects by soil microbes has not yet been reported.

In the present study, we aimed to evaluate the incidence of soil-borne diseases in farmlands by diagnosing soil biological characteristics. We examined a simple method to evaluate suppressiveness by soil microorganisms against a pathogen by co-cultivating indigenous soil microorganisms and a pathogenic fungus (*F. oxysporum* f. sp. *spinaciae*) because pathogenic *F. oxysporum* causes serious damage to various crops, and, thus, a feasible diagnostic method for diseases is required. We inoculated *F. oxysporum* onto the center of agar medium plates mixed with dilution series of a suspension of soil or an organic fertilizer. After cultivation, the sizes of *F. oxysporum* colonies were measured on the plates with and without the inoculation of the suspension, and the growth degree of *F. oxysporum* by the suspension was estimated. This method may be used to comprehensively evaluate the abundance, activity, and antagonistic ability of the soil microbial community because a smaller number of microbes in the diluted suspension may facilitate the proliferation of pathogens and distinct microbial compositions in the suspensions may provide different degrees of suppressiveness. After the evaluation of variations in the growth degree of *F. oxysporum* on the plates, we examined the growth degrees of *F. oxysporum* for organic fertilizers and soils applied with organic fertilizers. We also analyzed relationships between the growth degrees of *F. oxysporum* and disease incidence in spinach for soil inoculated with a pathogenic *F. oxysporum* strain in a pot experiment.

## Materials and Methods

### Experiment 1. Variations in estimated values of the growth degree of *F. oxysporum*

#### Soil

Soil samples were collected from a long-term experimental field with the application of organic fertilizers since 1986 at the Tsukuba Research Institute Farm, Katakura Chikkarin, Tsuchiura, Japan. The soil was Andosol. Soil samples were collected from a plot applied with rapeseed meal and stored at 4°C before the analysis. The chemical characteristics of soil are shown in Table S1.

#### Pathogenic fungal strain

*F. oxysporum* f. sp. *spinaciae* (IFO 6385) was used. It was cultivated on potato dextrose agar (potato extract 100 mL, glucose 20 g, and agar 15 g, distilled water 900 mL) at 30°C for 7 d. A square section with 5 mm each side of the fungal lawn was excised and used as an inoculum in subsequent experiments.

#### Co-cultivation of *F. oxysporum* with soil microorganisms

**Fig. 1:**
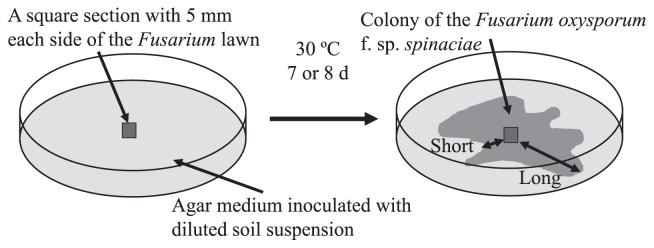
Scheme of the evaluation of colony development by *Fusarium oxysporum* f. sp. *spinaciae*.

Ten-gram portions of the soil sample were taken into a sterilized tube containing 90 mL of sterilized tap water and shaken reciprocally at 200 rpm for 30 min. One milliliter of the suspension was poured into 9 mL of sterilized tap water, mixed well, and serially diluted in the same manner. A dilution series was prepared to a magnification of 10^−6^ fold. A quantity of 1.0 mL of 10^−1^ to 10^−6^-fold diluted suspensions was inoculated into a petri dish and 16 mL of YPMG agar medium (Peptone-yeast extract medium; yeast extract 3 g, peptone 5 g, beef extract 3 g, glucose 10 g, agar 15 g, and distilled water 1 L; pH 7.0) (3) was poured and mixed in triplicate. A square section of the *F. oxysporum* hyphal lawn was placed in the center of agar medium (Fig. 1). As a control, sterilized water was inoculated instead of the diluted suspension of soil samples. The plates were incubated at 30°C for approximately one week (7 or 8 d), by which time the colony of *F. oxysporum* had spread fully on the control plate.

When sample suspensions were inoculated, the extension of *F. oxysporum* hyphae was generally inhibited by the growth of microorganisms in the sample on the plate. We used three kinds of calculation methods to evaluate the growth degree of *F. oxysporum* because the colony of *F. oxysporum* species was not always an entire circle. In order to estimate the area, we took a picture of the plate, printed it out and calculated the area of the colony of *F. oxysporum* by comparing the weight of the printed paper between the areas for the colony and whole plate. The ellipse area was considered to be an approximate area of the colony that was easy to evaluate. We measured the longest length of the area of hyphal growth as the longest diameter of the ellipse and also the shortest diameter that perpendicularly crossed the longest diameter at the center of the colony in order to calculate the area of the ellipse. The growth of antagonists in the soil suspension adjacent to the colony of *F. oxysporum* on the plates may have inhibited the extension of the mycelia of *F. oxysporum* and made the colony irregular in shape with a hollow (Fig. 1). Since this kind of antagonism is easy to evaluate at the place at which the extension of the mycelia of *F. oxysporum* was inhibited around the colony of the antagonist, the length of the shortest part of the colony together with the longest length was measured; *i.e.*, the extension of hyphae was measured at the parts at which the hyphae had grown the most (long diameter) and the least (short diameter) in the colony of *F. oxysporum* for soil samples and the control (Fig. 1), and the mean of these values was used to calculate the growth degree. As a representative value for the growth degree of *F. oxysporum*, the median of the estimated values of the growth degree at six dilutions from 10^−1^ to 10^−6^ was calculated.

### Experiment 2. Growth degree of *F. oxysporum* for organic fertilizers and soil applied with organic fertilizer

#### Soil

Commercial Andosol (“Kurotsuchi”, Joyful Honda, Tsuchiura, Japan) collected in Chiba prefecture was used to examine growth degrees for soil applied with organic fertilizers. The chemical characteristics of soil are shown in Table S1.

#### Organic fertilizer

Steamed bone meal (Katakura Chikkarin, Tokyo, Japan), a cow dung compost (“Soil Power 2”, Tokiwa Farm, Tsuchiura, Japan), and a microbial inoculant (“BIO-YUUKI”, Katakura Chikkarin, N-P_2_O_5_-K_2_O=3-3-3%) that was prepared with various waste from plants and animals such as crab shells, clay minerals, potassium silicate, and bacterial and fungal cultures were used. A compound inorganic fertilizer (“KUDOIRI-KUMIAI-Eco-KASEI 888”, Co-op Chemical, Tokyo, Japan, N-P_2_O_5_-K_2_O=8-8-8%) was used as a control. Steamed bone meal is an organic fertilizer that is used as a raw material for the production of organic fertilizers, but is mainly composed of calcium phosphate and exhibits almost no biological activity (17). Therefore, we used steamed bone meal as another control.

#### Pathogenic fungal strain

*F. oxysporum* f. sp. *spinaciae* (IFO 6385) was used.

#### Estimation of the growth degree of *F. oxysporum*

The growth degree of *F. oxysporum* was measured for the fertilizers tested (compound inorganic fertilizer, steamed bone meal, cow dung compost, and the microbial inoculant) by the co-cultivation of *F. oxysporum* with a suspension of the fertilizer that was prepared as described for soil in Experiment 1. In order to assess the growth degree for soil applied with fertilizers, all plots were fertilized with the compound inorganic fertilizer (N-P-K=150-150-150 kg ha^−1^). The application rate of organic fertilizers was as follows: 200 kg ha^−1^, 10,000 kg ha^−1^, and 2,000 kg ha^−1^ for steamed bone meal, cow dung compost, and the microbial inoculant, respectively. Mixed soils were placed for two weeks in a greenhouse with water supplied in order to prevent the soil surface from drying, which represented the soil condition before the seeding or transplanting of crops. Treated soil was stored at 4°C until used. We only measured the extension and oval area of the colonies of *F. oxysporum* in this experiment. As a representative value for the growth degree of *F. oxysporum*, a median of the estimated values of the growth degree at six dilutions from 10^−1^ to 10^−6^ was calculated.

### Experiment 3. Inoculation experiment of pathogenic *F. oxysporum* strain to spinach

#### Soil

A soil sample collected from a plot applied with compound inorganic fertilizer in the long-term experimental field described in Experiment 1 in March, 2015 was used. The chemical characteristics of soil are shown in Table S1.

#### Organic fertilizer

The same organic fertilizers as those in experiment 2 were used.

#### Pathogenic fungal strain

*F. oxysporum* f. sp. *spinaciae* (MAFF 103060) was used. This strain shows good reproducibility for the incidence of wilt disease in the inoculation experiment to spinach. It was cultivated in 100 mL of potato sucrose broth (potato extract [prepared from 1 kg potatoes boiled in 1 L of water] 100 mL, sucrose 20 g, distilled water 900 mL) at 30°C for 7 d by shaking horizontally. The number of conidiospores was enumerated on a hemocytometer and diluted culture solutions with a predetermined density were used for the inoculation.

#### Crop

Spinach (*Spinacia oleracea* L.) (“OKAME”, TAKII, Kyoto, Japan) was used.

#### Cultivation of spinach and inoculation of the pathogenic *F. oxysporum* strain

Spinach seeds pretreated in water for two d were sown into a cell tray (200 holes) filled with a nursery soil (“YASAI-BAIDO ICHI GOU”, Katakura Chikkarin) and grown in a greenhouse for one week. The conidiospores of *F. oxysporum* were added to the soil sample collected from the long-term experimental field at a dose of 10^6^ conidia g^−1^ soil and 500 mL of soil (approximately 400 g) was placed into polycarbonate pots (12 cm outer diameter×11.5 cm height; 0.01 m^2^). All plots were fertilized with the compound inorganic fertilizer (N-P-K=150-150-150 kg ha^−1^). The application rate of organic fertilizer was as follows: 200 kg ha^−1^ for steamed bone meal, 10,000 kg ha^−1^ for cow dung compost, and 2,000 and 10,000 kg ha^−1^ for the microbial inoculant. After 15 d of the application of the fertilizers to soil, three spinach seedlings were planted in each pot in triplicate on May 29, 2015. We noted the disease incidence and severity of wilting for each spinach plant on June 17, 2015 and collected soil samples from the pots in order to measure the growth degree of *F. oxysporum*. The incidence and severity of wilt were evaluated as follows: 0, healthy; 1, one leaf had wilted; 2, two or three leaves had wilted; 3, half of the leaves had wilted; 4, more than half of the leaves had wilted; 5, dead or nearly dead. We only measured the extension and oval area of the colonies of *F. oxysporum* in this experiment. As a representative value for the growth degree of *F. oxysporum*, a median of the estimated values of the growth degree at six dilutions from 10^−1^ to 10^−6^ was calculated.

#### Statistical analysis

All statistical tests were performed with Microsoft Excel 2013 for Windows (Microsoft, Tokyo, Japan) and Ekuseru-Toukei 2015 (Social Survey Research Information, Tokyo, Japan).

## Results

### Experiment 1. Variations in estimated values of the growth degree of *F. oxysporum*

**Fig. 2:**
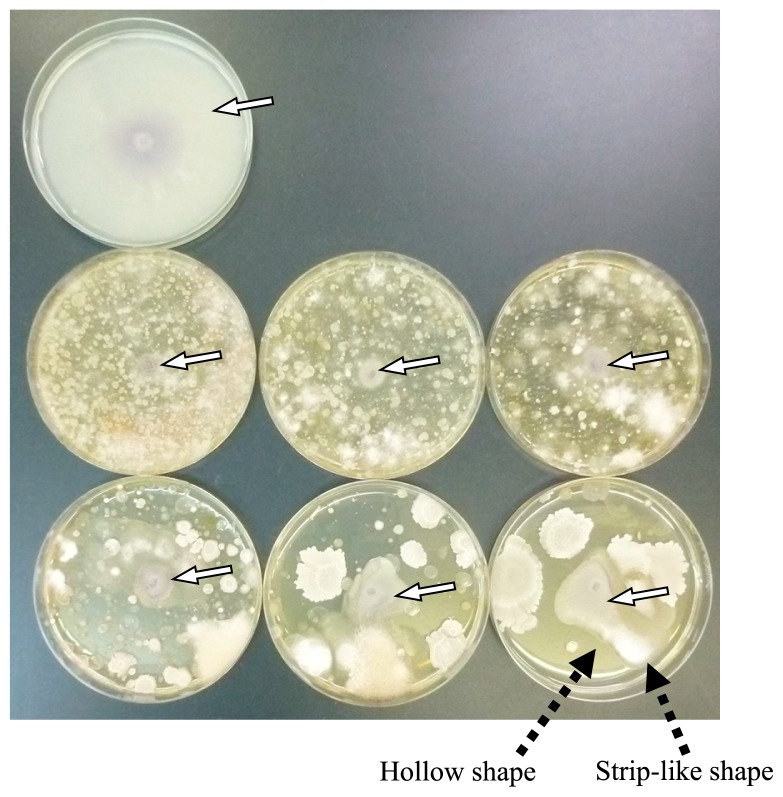
Colonies of *Fusarium oxysporum* f. sp. *spinaciae* on agar plates. A plate in the upper line, uninoculated; plates in the middle line, dilutions of 10^−1^, 10^−2^, and 10^−3^ from left to right; plates in the lower line, dilutions of 10^−4^, 10^−5^, and 10^−6^ from left to right. White arrows show colonies of *F. oxysporum* f. sp. *spinaciae* and black arrows indicate the strip-like shape and hollow shape of the colony.

**Fig. 3:**
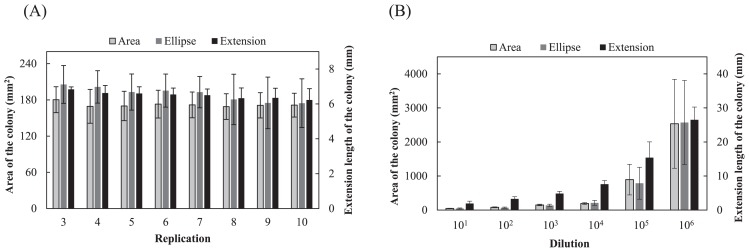
(A) Growth degree of *Fusarium oxysporum* f. sp. *spinaciae* based on an estimation of the real area, area as an ellipse, and extension length of the colony. The mean of medians of the degrees at dilutions from 10^−1^ to 10^−6^ for 3 to 10 plates in 10 replicates is shown with SD. (B) Growth degree of *F. oxysporum* f. sp. *spinaciae* at each dilution. Values show the mean of degrees with SD (*n*=10). The real area, ellipse area, and extension length for control plates were 5,256 mm^2^, 5,342 mm^2^, and 40.5 mm, respectively.

Almost no growth of *F. oxysporum* colonies was observed on plates with dilutions of soil from 10^−1^ to 10^−3^. Colonies of *F. oxysporum* that grew on plates with dilutions from 10^−4^ to 10^−6^ were circular or oval and some colonies showed a strip-like or hollow shape (Fig. 2). Fig. 3A shows the average growth degree estimated for 3 to 10 plates in 10 replicates. No significant difference was observed between the real area and ellipse area of colonies in 3 to 10 plates (*P*=0.124–0.821; *t*-test); however, both values showed large variations. Variations were markedly smaller for values of the extension length than the real and ellipse areas of colonies, for which the mean values of the coefficients of variation from 3 to 10 plates were 13.1%, 17.8% and 6.7% for the real area, ellipse area, and extension, respectively. Fig. 3B shows the mean values of the growth degree at every dilution, with 10 replicates. No significant differences were observed between the real area and ellipse area of colonies at each dilution from 10^−1^ to 10^−6^ (*P*=0.120–0.949; *t*-test); however, both values showed large variations. Variations were markedly smaller for the values of extension length than for the real and ellipse areas of colonies, for which the mean values of the coefficients of variation from 10^−1^ to 10^−6^ dilutions were 27.5%, 39.2%, and 21.2% for the real area, ellipse area, and extension, respectively. In the estimation of the degree based on the extension length of colonies, the degree increased gradually as the magnification of the dilution of the suspension increased, while it was maintained at almost the same level up to the 10^−4^ dilution and increased at the 10^−5^ and 10^−6^ dilutions with large variations in estimations based on the real and ellipse areas of colonies.

### Experiment 2. Growth degree of *F. oxysporum* for organic fertilizers and soil applied with organic fertilizers

**Fig. 4:**
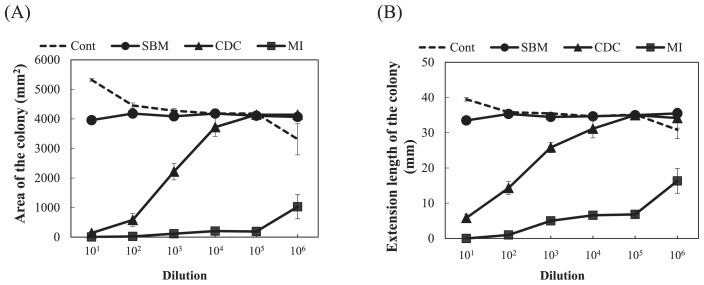
Growth degree of *Fusarium oxysporum* f. sp. *spinaciae* for organic fertilizers at each dilution based on an estimation of the ellipse area (A) and extension length (B) of the colony. Cont, compound inorganic fertilizer; SBM, steamed bone meal; CDC, cow dung compost; MI, microbial inoculant. Values show the mean of medians of degrees with SE (*n*=3). The ellipse area and extension length for control plates were 4,475 mm^2^ and 36.5 mm, respectively.

**Fig. 5:**
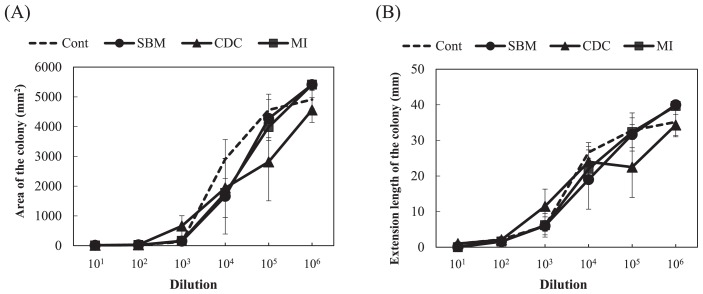
Growth degree of *Fusarium oxysporum* f. sp. *spinaciae* for soil applied with organic fertilizers at each dilution based on an estimation of the ellipse area (A) and extension length (B) of the colony. Cont, compound inorganic fertilizer; SBM, steamed bone meal; CDC, cow dung compost; MI, microbial inoculant. Values show the mean of medians of degrees with SE (*n*=3). The ellipse area and extension length for control plates were 5,473 mm^2^ and 40.5 mm, respectively.

The growth degrees of *F. oxysporum* were significantly smaller for the microbial inoculant and cow dung compost than for the control (compound inorganic fertilizer) in the estimations based on both extension length and ellipse area (*P*<0.05, Dunnett’s test) (Fig. 4). The degrees for steamed bone meal were not significantly different from those for the control. The growth degree for the microbial inoculant was maintained at low values even at a high magnification of dilutions, while it was only low at the low magnification of dilutions and then significantly increased in the diluted samples for cow dung compost (Fig. 4). The representative values of the growth degrees, calculated as the median of the degrees at all dilutions from 10^−1^ to 10^−6^, for cow dung compost and the microbial inoculant were significantly lower than the value obtained for the control (*P*<0.01, Dunnett’s test) (Table 1). The representative value for steamed bone meal was not significantly different from that for the control. Large variations were observed and no significant differences were noted in growth degrees among soil applied with fertilizers (Fig. 5). The representative values of the degrees showed no significance among the soil samples tested (*P*>0.05, Dunnett’s test) (Table 1).

### Experiment 3. Inoculation experiment of the pathogenic *F. oxysporum* strain to spinach

**Fig. 6:**
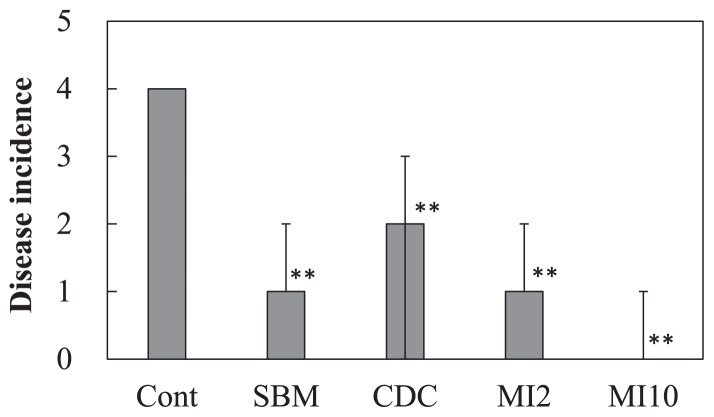
Disease incidence of spinach by *Fusarium oxysporum* f. sp. *spinaciae* in each plant. Cont, compound inorganic fertilizer; SBM, steamed bone meal; CDC, cow dung compost; MI2, microbial inoculant applied with 2,000 kg ha^−1^; MI10, microbial inoculant applied with 10,000 kg ha^−1^. Values show the median with the upper and lower quartile points (*n*=9). ** indicates a significant difference from the control plot (*P*<0.01, Steel test).

**Fig. 7:**
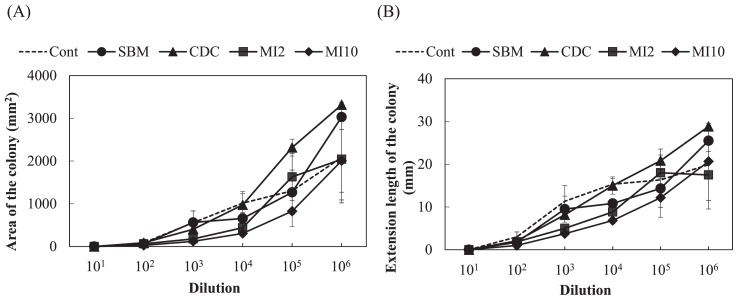
Growth degree of *Fusarium oxysporum* f. sp. *spinaciae* at each dilution based on an estimation of the ellipse area (A) and extension length (B) of the colony. Cont, compound inorganic fertilizer; SBM, steamed bone meal; CDC, cow dung compost; MI2, microbial inoculant applied with 2,000 kg ha^−1^; MI10, microbial inoculant applied with 10,000 kg ha^−1^. Values show the mean of medians of degrees with SE (*n*=3). The ellipse area and extension length for control plates were 3,404 mm^2^ and 29.7 mm, respectively.

**Fig. 8:**
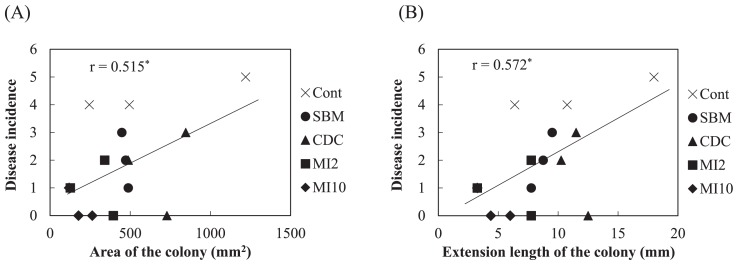
Correlation between the disease incidence in spinach by *Fusarium oxysporum* f. sp. *spinaciae* in each plant and the mean of median values of growth degrees of *Fusarium oxysporum* f. sp. *spinaciae* on an estimation of the ellipse area (A) and extension length (B) of the colony (*n*=15). * indicates a significant difference (*P*<0.05).

In the pot experiment with spinach, a large number of leaves wilted in the control plot applied with the compound inorganic fertilizer, while few leaves wilted in the microbial inoculant plot with 10,000 kg ha^−1^ (Supplemental Fig. S1). Fig. 6 shows the disease incidence among spinach plants. The disease incidence was significantly lower with the application of organic fertilizers than with the compound inorganic fertilizer (*P*<0.01, Steel test). The growth degrees of *F. oxysporum* for soils estimated based on the extension length and ellipse area of colonies are shown in Fig. 7. Large variations were observed and no significant difference was noted in the growth degrees among soils based on the ellipse area of colonies. On the other hand, the growth degrees for the microbial inoculant plot with 10,000 kg ha^−1^ were slightly lower than that for the control plot in the estimation based on the extension length of colonies, but with a significance level of 10% (*P*=0.063, Dunnett’s test). The representative values of the growth degrees based on the extension length of the colonies for the microbial inoculant plot with 10,000 kg ha^−1^ were significantly lower than that for the control plot (Table 1; *P*<0.05, Dunnett’s test), and the value based on the ellipse area for the plot with 10,000 kg ha^−1^ was slightly lower than that for the control plot (Table 1; *P*=0.068). Correlations (*P*<0.05) were found between the disease incidence and growth degrees based on the extension length and ellipse area of colonies (Fig. 8).

## Discussion

We herein described a culture method to measure the sizes of *F. oxysporum* colonies on agar plates that does not require any special apparatus. Therefore, the present method has priority over that by Hashimoto *et al.* (9), which also evaluates the proliferation of pathogens in soil suspension, but requires a biosensor.

In the measurement of the area of *F. oxysporum* colonies, an estimation of the area as an ellipse may represent a simple alternative to the measurement of a real area because no significant difference was found in growth degrees based on both estimations (Fig. 3). Variations in the growth degree of *F. oxysporum* were smaller in the estimation based on the extension length than that based on the real and ellipse areas of *F. oxysporum* colonies (Fig. 3A). In the estimation of the extension length of colonies, the shortest and longest diameters were measured and the mean of the values was used for the evaluation, which may have reduced variations in the values of the degrees to less than that with the use of squared values in the estimation of real and ellipse areas. Differences in the increases observed in degrees among estimations as the magnification of dilutions increased in Fig. 3B may also have due to the same reason. A marked rise in growth degrees at the 10^−5^ and 10^−6^ dilutions for the estimation based on the extension length indicate the practical superiority of this estimation to detect small changes in growth degrees (Fig. 3B). It may be possible to evaluate a slight difference at a low magnitude of dilutions of a soil suspension. The median value of the growth degrees at all dilutions from 10^−1^ to 10^−6^ seemed to exhibit well the rising trends of the degrees as increase in the magnification of dilution as a whole and thus was used as a representative of the degrees.

Increases in microbial diversity in soil and the proliferation of antagonistic microbes against pathogens have been suggested to cause disease suppressiveness by soil (12). In the present method, diverse microorganisms may have caused suppressiveness when the shape of the colonies was a concentric circle, while specific microbes that are antagonistic to pathogens may have contributed to suppressiveness when the colonies had a hollow shape (Fig. 2). Regarding the assumption described in the Materials and Methods section, colonies in an irregular shape with a hollow were observed, and may have been caused by the growth of antagonists beside the hyphae of *F. oxysporum* on the plate (Fig. 2). When the growth degree was evaluated based on the ellipse area of the colony, this effect by antagonists in indigenous microorganisms in a soil suspension may have been ignored. Therefore, an estimation of the extension length of the colonies is advantageous. However, it is important to note that the scattering of specific microbes grown on plates may alter the values of the growth degree, particularly at a high magnification of dilutions.

In order to use the growth degree of *F. oxysporum* as a biological diagnostic index of soil, the relationship between the growth degree and disease incidence in plants by pathogenic *F. oxysporum* needs to be clarified. El-Mohamedy *et al.* (8) showed the different suppressive degrees of *Fusarium* dry rot of potato for composts prepared from various kinds of plant residues inoculated with useful bacterial strains. The suppressive degrees of cow dung compost and the microbial inoculant were markedly higher than that of the compound inorganic fertilizer (Table 1 and Fig. 4). However, soil applied with these organic fertilizers did not show marked differences in the suppressive degree from control soil applied with the compound inorganic fertilizer (Table 1 and Fig. 5). This may be because the application rates of the fertilizers were less than 1% of the amount of soil and the soil itself exhibited suppressiveness derived from indigenous microorganisms. In the inoculation experiment of pathogenic *F. oxysporum* to spinach, the application of organic fertilizers decreased the disease incidence (Fig. 6), and the growth degree of *F. oxysporum* colonies was significantly lower for the microbial inoculant plot with 10,000 kg ha^−1^ than the other plots (Table 1). In addition, a correlation was found between the disease incidence and representative value of the growth degrees of *F. oxysporum* colonies calculated as the median of the degrees at all dilutions (Fig. 8). These results indicate that the estimation of the growth degree of *F. oxysporum* by the present method is applicable to the biological diagnosis of soil suppressiveness against a pathogen. However, steamed bone meal and cow dung compost significantly decreased the disease incidence (Fig. 6), while the growth degrees of *F. oxysporum* colonies were not significantly different from that for the control plot. This inconsistency suggests that other factors such as improvements in soil chemical properties, which cannot be evaluated by the co-culture method in the present study, contributed to the disease incidence in the inoculation experiment. This needs to be considered when applying this method. Further investigations are needed in order to elucidate the relationship between disease incidence in plants and the growth degrees of pathogenic *F. oxysporum* by this method for suppressive and conducive soils under the long-term application of inorganic and organic fertilizers and for various combinations of crops and pathogenic *F. oxysporum*.

## Conclusion

Measurements of the growth degree of *F. oxysporum* in this study may represent a simple method that provides useful information on the suppressiveness of organic fertilizers and soil against soil-borne diseases by pathogenic *F. oxysporum*.

## Supplementary Information



## Figures and Tables

**Table 1:** Mean of median values of growth degrees of *Fusarium oxysporum* f. sp. *spinaciae* at dilutions from 10^−1^ to 10^−6^ with SE (*n*=3)

Fertilizer	Experiment 2 Organic fertilizer		Soil applied with fertilizer		Plot	Experiment 3 Soil planted with spinach	
		
	Ellipse area (mm^2^)	Extension length (mm)	Ellipse area (mm^2^)	Extension length (mm)		Ellipse area (mm^2^)	Extension length (mm)
Cont	4249 ± 66	35.3 ± 0.3	1420 ± 259	15.3 ± 0.9	Cont	653 ± 292	11.7 ± 3.4
SBM	4104 ± 68	34.9 ± 0.1	784 ± 553	11.7 ± 4.6	SBM	469 ± 17	8.7 ± 0.5
CDC	2960 ± 288 ^**^	27.5 ± 1.6 ^**^	689 ± 259	13.5 ± 4.7	CDC	688 ± 106	11.4 ± 0.7
MI	143 ± 6 ^**^	5.6 ± 0.1 ^**^	967 ± 44	14.2 ± 1.8	MI2	287 ± 82	6.3 ± 1.5 †
					MI10	187 ± 42 †	4.5 ± 0.8 ^*^

* and ** indicate significant differences from the control at the 5% and 1% levels (*P*<0.05 and *P*<0.01; Dunnett’s test), respectively;

† indicates the 10% level (*P*<0.1; Dunnett’s test).

Cont, control (compound inorganic fertilizer); SBM, steamed bone meal; CDC, cow dung compost; MI, microbial inoculant; MI2, microbial inoculant applied with 2,000 kg ha^−1^; MI10, microbial inoculant applied with 10,000 kg ha^−1^.
