# An Evaluation Method for Suppression of Pathogenic *Fusarium oxysporum* by Soil Microorganisms Using the Dilution Plate Technique

**DOI:** 10.1264/jsme2.ME16052e

**Published:** 2017-06

**Authors:** Masahiro Mitsuboshi, Yuuzou Kioka, Katsunori Noguchi, Susumu Asakawa

**Affiliations:** 1 Tsukuba Research Institute, Katakura & Co-op Agri Corporation5–5511 Namiki, Tsuchiura 300–0061Japan; 2 Katakura & Coop Agri Corporation1–8–10 Kudankita, Chiyoda, Tokyo 102–0073Japan; 3 Graduate School of Bioagricultural Sciences, Nagoya University1 Furo-cho, Chikusa, Nagoya 464–8601Japan

Volume 31, No. 3, Page 307–313, 2016

Page 310, Legend for Fig. 4

Incorrect

**Fig. 4.** Growth degree of *Fusarium oxysporum* f. sp. *spinaciae* for organic fertilizers at each dilution based on an estimation of the ellipse area (A) and extension length (B) of the colony. Cont, compound inorganic fertilizer; SBM, steamed bone meal; CDC, cow dung compost; MI, microbial inoculant. Values show mean of medians of degrees with SE (*n*=3). The ellipse area and extension length for control plates were 4,475 mm^2^ and 36.5 mm, respectively.

Correct

**Fig. 4.** Growth degree of *Fusarium oxysporum* f. sp. *spinaciae* for organic fertilizers at each dilution based on an estimation of the ellipse area (A) and extension length (B) of the colony. Cont, compound inorganic fertilizer; SBM, steamed bone meal; CDC, cow dung compost; MI, microbial inoculant. Values show mean of growth degrees with SE (*n*=3). The ellipse area and extension length for control plates were 4,475 mm^2^ and 36.5 mm, respectively.

Page 311, Legend for Fig. 5

Incorrect

**Fig. 5.** Growth degree of *Fusarium oxysporum* f. sp. *spinaciae* for soil applied with organic fertilizers at each dilution based on an estimation of the ellipse area (A) and extension length (B) of the colony. Cont, compound inorganic fertilizer; SBM, steamed bone meal; CDC, cow dung compost; MI, microbial inoculant. Values show the mean of medians of degrees with SE (*n*=3). The ellipse area and extension length for control plates were 5,473 mm^2^ and 40.5 mm, respectively.

Correct

**Fig. 5.** Growth degree of *Fusarium oxysporum* f. sp. *spinaciae* for soil applied with organic fertilizers at each dilution based on an estimation of the ellipse area (A) and extension length (B) of the colony. Cont, compound inorganic fertilizer; SBM, steamed bone meal; CDC, cow dung compost; MI, microbial inoculant. Values show the mean of growth degrees with SE (*n*=3). The ellipse area and extension length for control plates were 5,473 mm^2^ and 40.5 mm, respectively. Page 312, Legend for Fig. 7

Incorrect

**Fig. 7.** Growth degree of *Fusarium oxysporum* f. sp. *spinaciae* at each dilution based on an estimation of the ellipse area (A) and extension length (B) of the colony. Cont, compound inorganic fertilizer; SBM, steamed bone meal; CDC, cow dung compost; MI2, microbial inoculant applied with 2,000 kg ha^−1^; MI10, microbial inoculant applied with 10,000 kg ha^−1^. Values show the mean of medians of degrees with SE (*n*=3). The ellipse area and extension length for control plates were 3,404 mm^2^ and 29.7 mm, respectively.

Correct

**Fig. 7.** Growth degree of *Fusarium oxysporum* f. sp. *spinaciae* at each dilution based on an estimation of the ellipse area (A) and extension length (B) of the colony. Cont, compound inorganic fertilizer; SBM, steamed bone meal; CDC, cow dung compost; MI2, microbial inoculant applied with 2,000 kg ha^−1^; MI10, microbial inoculant applied with 10,000 kg ha^−1^. Values show the mean of growth degrees with SE (*n*=3). The ellipse area and extension length for control plates were 3,404 mm^2^ and 29.7 mm, respectively. The authors would like to apologize for these corrections and any inconvenience caused.

