# Supplementary material for: An Evaluation Method for the Suppression of Pathogenic Fusarium oxysporum by Soil Microorganisms Using the Dilution Plate Technique
Source: Microbes Environ. 2016 Aug 24;31(3):307–13. doi: 10.1264/jsme2.ME16052 (PMC5017808; doi:10.1264/jsme2.ME16052)
Supplement: Supplementary file 1 [file 31_307_s1.pdf]

Table S1. Chemical characteristics of soil used in experiments in the present study[a]

	Experiment 1	Experiment 2	Experiment 3
pH (H_2O)	6.86	5.95	7.09
EC (dS m^{-1})	0.11	0.12	0.08
NH_3-N (mg kg^{-1})	8.5	6.7	9.5
NO_3-N (mg kg^{-1})	14.8	26.9	7.4
Available P (P_2O_5 mg kg^{-1})[b]	1,940	20	30
Exchangeable K (K_2O mg kg^{-1})	490	280	300
Exchangeable Ca (CaO mg kg^{-1})	3,980	3,310	3,040
Exchangeable Mg (MgO mg kg^{-1})	680	540	770
Phosphate absorption coefficient	2565	2824	2662
CEC ($cmol_c$ kg^{-1})	22.2	27.7	20.8

[a] pH and EC were measured in moist soil and the other characteristics were measured in air-dried soil.

[b] Truog method.

Fig. S1 Growth of spinach on June 17, 2015. Cont, compound inorganic fertilizer; MI10, microbial inoculant applied with 10,000 kg ha^{-1}.

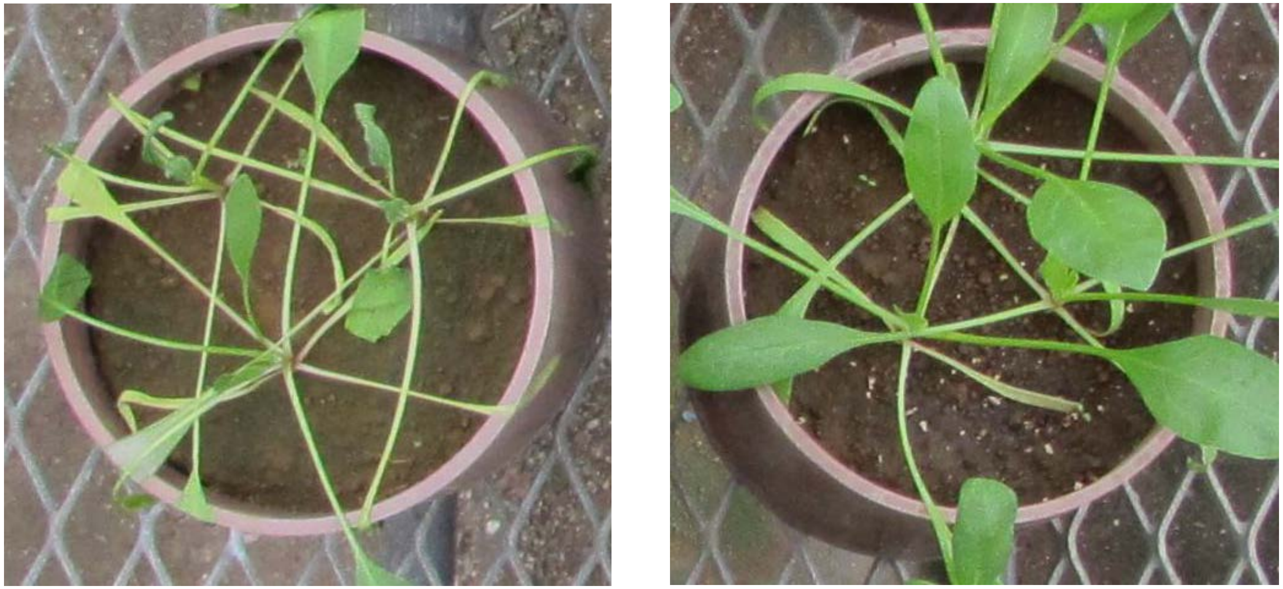

Cont MI10

Fig. S1 Mitsuboshi
